# An informatics model for guiding assembly of telemicrobiology workstations for malaria collaborative diagnostics using commodity products and open-source software

**DOI:** 10.1186/1475-2875-8-164

**Published:** 2009-07-17

**Authors:** West Suhanic, Ian Crandall, Peter Pennefather

**Affiliations:** 1 Laboratory for Collaborative Diagnostics, Leslie Dan Faculty of Pharmacy, University of Toronto, 144 College St, Toronto, Ontario, M5S 2S2, Canada; 2 Department of Laboratory Medicine and Pathobiology, University of Toronto, Toronto, Ontario M5S 1A8, Canada; 3 McLaughlin-Rotman Centre/University Health Network, 101 College St, Toronto, Ontario, M5G 1L7, Canada

## Abstract

**Background:**

Deficits in clinical microbiology infrastructure exacerbate global infectious disease burdens. This paper examines how commodity computation, communication, and measurement products combined with open-source analysis and communication applications can be incorporated into laboratory medicine microbiology protocols. Those commodity components are all now sourceable globally. An informatics model is presented for guiding the use of low-cost commodity components and free software in the assembly of clinically useful and usable telemicrobiology workstations.

**Methods:**

The model incorporates two general principles: 1) collaborative diagnostics, where free and open communication and networking applications are used to link distributed collaborators for reciprocal assistance in organizing and interpreting digital diagnostic data; and 2) commodity engineering, which leverages globally available consumer electronics and open-source informatics applications, to build generic open systems that measure needed information in ways substantially equivalent to more complex proprietary systems. Routine microscopic examination of Giemsa and fluorescently stained blood smears for diagnosing malaria is used as an example to validate the model.

**Results:**

The model is used as a constraint-based guide for the design, assembly, and testing of a functioning, open, and commoditized telemicroscopy system that supports distributed acquisition, exploration, analysis, interpretation, and reporting of digital microscopy images of stained malarial blood smears while also supporting remote diagnostic tracking, quality assessment and diagnostic process development.

**Conclusion:**

The open telemicroscopy workstation design and use-process described here can address clinical microbiology infrastructure deficits in an economically sound and sustainable manner. It can boost capacity to deal with comprehensive measurement of disease and care outcomes in individuals and groups in a distributed and collaborative fashion. The workstation enables local control over the creation and use of diagnostic data, while allowing for remote collaborative support of diagnostic data interpretation and tracking. It can enable global pooling of malaria disease information and the development of open, participatory, and adaptable laboratory medicine practices. The informatic model highlights how the larger issue of access to generic commoditized measurement, information processing, and communication technology in both high- and low-income countries can enable diagnostic services that are much less expensive, but substantially equivalent to those currently in use in high-income countries.

## Background

### A global deficit in laboratory medicine infrastructure

Deficits in medical infrastructure are widely recognized as hindering coordinated responses to global health challenges in low-income countries [1-3]. More specifically, deficits in laboratory medicine resources and expertise limits the ability of many developing countries to perform adequate evaluations of new and potentially transformative diagnostic practices. It also hinders their ability to diagnose and evaluate need, quality, and safety of existing practices [4]. It is suggested here that important determinants of the origins of these deficits and possible solutions are related to the level of development of locally available measurement [M] and communication [C] technologies (Table 1). Efforts to correct deficits in local laboratory medicine capacity may be able to leverage concerted global health efforts to deal with global pandemics like malaria. These global health efforts require a capacity to monitor the performance and quality of interventions and that capacity in turn is reliant on deploying measurement and communication technologies.

**Table 1:** Laboratory infrastructure deficit origins and solutions (modified from [2])

**Deficit Origins**	**Potential solutions**
Lack of laboratory consumables [M]	Emphasize importance of laboratory testing [C]

Lack of basic essential equipment [M]	Balance the allocation of financial resources [M]

Limited numbers of skilled personnel [C]	Establish system for laboratory accreditation [C]

Lack of educators and training programs [C]	Implement laboratory training programs [C]

Inadequate logistical support [M, C]	Routinely monitor test quality [M]

Insufficient monitoring of test quality [C, M]	Encourage public/private partnerships [C]

Decentralization of laboratory facilities [C]	Develop affordable, rapid diagnostic tests [M]

Lack of governmental standards [C]	Strengthen existing healthcare structure [M]

M = Measurement related, C = Communication related

### Telediagnostics and telemicroscopy

Telemedicine was originally conceived as the use of TV technology to enable remote examination of patients by physicians [3]. However, over the last half of the 20^th^-century and the first decade of the 21^rst ^century, results of clinical laboratory tests have become essential complements to traditional clinical evaluation of physical signs and symptoms in arriving at a diagnosis of the patient's condition. A simple physical examination is no longer sufficient to guide care. Indeed, the core diagnoses of certain disease states, of relevance for global health, are now often defined primarily in terms of laboratory tests. A good example is the use of CD4 flow cytometry for defining HIV/AIDS [5]. In parallel, there have been tremendous changes in technology used for recording, interpreting, and communicating laboratory test results. Most diagnostic test results, and especially those tests involving various forms of macroscopic and microscopic imaging, are captured, interpreted, and communicated digitally.

This general trend in medicine of defining disease in terms of digital, and often image-based diagnostic data has stimulated development of certain telemedicine practices such as teleradiology and telepathology. These practices have been shown to reduce duplication costs and provide new opportunities for quality control. This in turn increases efficiency and effectiveness of standard practices [6-9]. Although image-based test results are also central to the diagnosis and effective treatment of infectious diseases including malaria [10,11], parallel development of telemicrobiology has been less significant [12]. Nevertheless, Linder *et al *[13] have recently demonstrated the potential utility of applying web-based virtual microscopy approaches, such as are used in telepathology, for parasitology education and quality assessment.

### Malaria diagnostic constraints

Diagnoses of parasitic infections rely heavily on direct observation of stool and blood smears using light microscopy [10,13]. To diagnose malaria, a trained microbiologist is expected to examine a stained blood smear with a microscope and detect as few as 10 parasites per microliter of blood as well as determine the species of *Plasmodium *that is present. This is an acquired and challenging skill, as the parasites are small (~1–2 μm), are present in a field of blood cells that is visually distracting, and many artifacts that resemble malaria parasites may also be present in the sample [10]. Proficiency needs to be maintained through structured training and frequent experience with the diagnostic task. Because failure to successfully perform that task can have serious consequences, there is a continuous need for oversight by experienced mentors and performance evaluators. Reliance on experienced microbiology experts for diagnosis, training and quality assessment often is problematic since such experts are increasingly in short supply and not being replaced following retirement [14,15].

Telemicrobiology could play an important role, both in enabling development of local capacity to interpret malaria blood smears and in distributing the diagnostic burden. In addition, a system-wide view of highly diverse, population-based diseases like malaria becomes available through shared access to results via telemicrobiology networks. As a result, local variations in disease presentation can be better accommodated, and limited clinical resources used more efficiently and effectively.

Investments in "Rolling Back Malaria" efforts and the need for microscopy-based malaria diagnostics to monitor effectiveness of those efforts, provides an opportunity to invest in the establishment of telemicrobiology infrastructure in low-income countries, where malaria often is endemic. Such efforts can create infrastructure that could also support many other types of microscopy-based laboratory medicine tests important for microbiological, haematological, and oncological diagnostics and begin to reverse the laboratory medicine deficits that are hindering health system performance in those countries. The key to success will be to develop an informatics model that is not only locally sustainable and supportable, but is also connected to and synchronized with global biomedical informatics resources and external quality assessment programs. This methodology paper formulates such a model using two new concepts: 1) commodity engineering and 2) collaborative diagnostics. The paper validates the informatic model through the design and assembly of a telemicrobiology workstation built of widely available commodity components that can operationalize the model.

### Formulation of the informatics model

#### Collaborative diagnostics enabled by distributed digital diagnostic data

In the traditional analog world of diagnostics, much of the supporting information about the diagnostic event is not present in final reports concerning that event. The establishment of new digital diagnostic systems has created new opportunities for digital recording, sharing, and analysis of health information in ways that spans multiple events. This in turn creates new opportunities to analyze the process of diagnostic information flow and the information ecology in which it operates. It also facilitates monitoring of the quality and fidelity of the diagnostic process. The ease of accessing and sharing digital information in turn is driving the development of collaborative health informatics.

This new information ecology enables network-based collaborative diagnostics, which is defined here as: *a participatory process enabled by the integration of multiple monitoring and communication devices with computer networks. The process involves multiple steps and people working together to carry out at least six stages of the diagnostic event: initiation, sensing, analysis, diagnosis, reporting, and indexing, It involves diagnostic procedures that generate digital results in a networked environment where transactions can be further transcribed, annotated, aggregated, indexed and reinterpreted at a later time for quality assurance and reflective analysis*.

Collaborative diagnostics is a complex idea. It is simultaneously: 1) a design constraint; 2) a testing method; 3) a diagnostic strategy; and 4) a means of managing adaptation to new and unanticipated constraints and situations. Despite this subtle complexity, collaborative diagnostics is useful as a mechanism for easing the constraints imposed by what Simon has termed bounded rationality [16], i.e. the limitations on rational action within complex systems imposed by time and capacity constraints. More agents participating in the sensemaking at the core of the diagnostic process means more diagnostic resources.

#### Commodity engineering of devices supporting collaborative diagnostics

Convergence and standardization of consumer electronics technology for digital recording (photography, music), communication (cell phones, Internet), data processing (PDA's, computers) and display (panels and projectors) are transforming the way observations can be recorded, interpreted, and communicated. Emerging standards in digital collaborative communication technology are enabling reliable and accurate transmission of information using globally accessible commodity devices. High-definition digital cameras that were previously distributed through specialty shops or scientific equipment companies are now widely used and available as consumer electronic commodities anywhere in the world.

The regime of widespread availability of commodity products that are useable by the general consumer, even in low-income countries, where technical support may be limited, raises the possibility of establishing clinical laboratory information management systems based on commodity components. This methodology paper illustrates how that aim can be accomplished through a commodity engineering approach of assembling engineered artifacts out of such commodity components. Commodity components are defined as those types of components that are: 1) distributed with low profit margins, 2) easy to source from multiple providers, 3) easy to replace, and 4) easy to substitute away from. These are components for which there are several substantially equivalent alternatives and sources. They can also be considered as FAIR components as they are: 1) flexible, 2) readily available, 3) inexpensive, and 4) easily replaceable. Examples of commodity computation and electronic equipment include: computer memory, computer motherboards, CPUs, disk drives, LCD flat panel displays, point-and-shoot cameras, cell phones etc. These components are available via a globalized supply chain in Montreal, Moscow, Maputo, Manila, or Montevideo. This global supply chain also functions as a just-in-time support network because if a component fails it can be easily replaced within hours or days. Another element critical to the approach is reliance on free and open-source software. Open-source software can be considered a commodity component for the reason that it: 1) is globally available; 2) has a global support network provided by the globalized network of programmers who developed the software; 3) is available for usually zero cost as a public good where individual use does not limit use by others. Likewise, use of commodity components necessarily makes the technology platform more open, generative, and adaptable to local constraints.

### Model description

#### Design constraints for building a telemicroscopy workstation

There are three key steps in implementing a telemicroscopy workstation using commodity engineering and collaborative diagnostics design principles: 1) sourcing and connecting a commodity digital camera to a commodity microscope in a way that generates an image of sufficient resolution that remote viewing is adequate for uncompromised expert interpretation of the microbiology sample; 2) creating the digital image processing and communication infrastructure that enable local measurements to be analysed and communicated in a distributed manner using commodity components and open-source software; and 3) creating a globally accessible and locally controllable picture archiving and communication system (PACS) using commodity components and open-source software. Each of these steps now can be accomplished in a variety of substantially equivalent ways.

#### Commoditized high-resolution digital telemicroscopy

Since a key design constraint is that the use of commodity components should not compromise the ability to recognize important morphologic and phenotypic details in the image, it is instructive to examine the physical and biophysical constraints limiting high resolution digital microscopy using commodity components.

The principles of microscopy have been explored over the last few centuries and are well described in online resources like Nikon's MicroscopyU. Maximum microscopy resolution is given by the formula 1.22λ/(NA objective + NA condenser), where λ represents the wavelength of light and NA is the numerical aperture of the microscope objective or condenser. For example, with epifluorescent illumination, where the objective also plays the role of condenser, the maximum possible resolution for 500 nm light and a 1.4 NA immersion objective (upper NA limit) is, therefore, around 0.22 μm. This calculation is independent of magnification, but magnification does come into play with respect to the resolving power of the detector.

When the human eye is the detector, maximal useful total magnification (i.e. Objective × Ocular magnification) is no greater than about 600–1200 times the objective's NA. This results from two biophysical constraints: 1) normal visual acuity being rated at between 0.5 and 1 arc-minute and 2) 50% of the retinal cells are located in the retinal fovea and pay attention to only a 10 degrees field of view (e.g. [10° × 60 arc-minutes/degree]/[(0.5–1)arc-minute] = 600–1200). For example, with a 1.4 NA, 100× immersion objective, a 10× ocular lens would not do justice to the resolving potential of the objective while a 20× ocular will deliver "empty magnification".

When a digital camera is used as the detector, a simple resolution rule is that a minimum of two pixels need to be addressed to unambiguously localize a feature in the image. This reflects the Nyquist-Shanon rule for digital resolution. To efficiently match the maximal theoretical physical resolution of 0.22 μm obtainable with 1.4 NA 100 × objectives, the detector pixel size should thus be at most 11 μm. To efficiently match the maximal resolution of 0.61 μm from a 0.5NA 16× objective the detector pixel size should be at most 4.5 μm. Multi-megapixel "point-and-shoot" cameras currently available for $200–$400 have 2–4 μm pixel spacing. Many of these are now available for less than $100 second hand. Furthermore, some point and shoot cameras, (e.g. Canon A series), have bayonet ridges that allow simple coupling to standard microscope camera adapters. These cameras can also the controlled through the USB data port using open-source camera control software applications (e.g. gPhoto). Thus, provided there is also access to data obtained using standardized target slides, results using many different assemblies of cameras, microscopes and objectives can be directly compared.

In some ways, given the simple bright or dark field imaging task presented to the camera for malaria diagnosis, consumer point-and-shoot cameras may be better choices for such systems than expensively customized photomicrograph systems. Paradoxically, producing a custom machined adapter containing a simple lens pair to focus the microscope image onto the camera sensor can cost as much as the mass produced camera it is adapted to. This is where local sourcing and manufacture of non-commodity components needed to integrate commodity components into the system can reduce overall costs.

Although the digital microscopy microbiology workstation can function usefully without Internet access, the ability to access remote microbiology expertise enabled by this design is invaluable. For remote collaborative diagnostic, training, and quality assessment applications, it is important to ensure that the remotely displayed digital image is equivalent to what could be observed through a microscope locally. It is useful, therefore, to consider what limitations the digital display resolution and the distance of the viewer from the screen might place on this requirement. The effectiveness of a computer monitor display again is influenced by human visual acuity. The human capacity to discriminate 1 arc-minute corresponds to an ability to resolve spots with diameters of 0.07 mm at an optimal viewing distance of 25 cm. Because of the way the eye integrates information concerning lines most people can easily resolve 20 line pairs per mm at 25 cm. This corresponds to about 1000 dots per inch (DPI) in printed material and 500 pixels per inch (PPI) per inch on a monitor. In general, human observers are well satisfied with 600 DPI print quality.

Typical LCD computer monitors have pixel pitches of around 0.25 mm depending on aspect ratios and this corresponds to a value of around 72–100 PPI. Although some newer notebook displays have as much as 150 PPI display resolution, normal human visual acuity is much higher and information can be lost. Small cell phone displays (2–4" diagonals) can have higher display resolution. These are currently manufactured with a typical display resolution of about 200 PPI (e.g the iPhone), but, higher resolution screens are anticipated soon. Still smaller (0.5" diagonals) VGA (640 × 480) and SVGA screens (800 × 600) used in electronic viewfinders that can be found in certain consumer cameras have resolutions of 1000 PPI resolution. These are becoming available commercially as commodity items. It is now possible to use mass produced consumer electronic components to create a digital "heads-up" microscope eye piece that satisfactorily reproduces what is seen through the ocular of an optical microscope.

The next element of the microscope system that can be commoditized is the illuminator. There have been a number of demonstrations that commodity high-power, solid-state, monochromatic, light-emitting-diodes (LEDs) can effectively replace specialty lasers and arc lamps traditionally used in microscope based microbiological diagnostic methods reliant on fluorescent dyes [17,18]. There has also been a recent suggestion that low resolution imaging of suspended arrays of malaria-infected blood cells illuminated using LED sources can be used to create flow cytometry-like data, equivalent to that obtainable using much more expensive systems [19]. Likewise a recently introduced innovative design [20] enables a Makler-type LED illuminator with built-in dichroic and excitation filter to be inserted into the objective body. This further simplifies the microscope light path and the cost of delivering epifluorescence microscopy (around $1500). These illuminators are not yet commodities. But, LED's are commodities and continue to become more powerful and less expensive. Another LED illuminator adaptor design has recently been introduced by Zeiss in conjunction with the Foundation for Innovative diagnostics (FIND) that attaches to their low-end Primo-Star line of microscopes. The Primo Star iLED and is being distributed at cost to low income countries where TB is endemic. The Partec CyScope is another example of a robust LED-based field microscope. Further, because of digital focusing and if the eye binocular attachments and ocular eye lens can be replaced by a digital camera, a significant saving can be realized by purchasing standard modular microscopes and this can offset the cost of the special microscope camera adapter.

#### Commoditized infrastructure that enables locally measured digital microscopy images to be analysed and discussed in a distributed manner

The next step after capturing a digital image with a camera is to transfer that information onto a local processing node where it can be annotated and examined and also shared via the internet or even physical transportation of storage media from one location to another. Commodity devices make possible a variety of collaborative diagnostic environments that differ by: 1) type (asynchronous/synchronous or real-time); 2) quality (low bit rate/high bit rate); 3) number of participants-(two to hundreds); and 4) media types-(video, audio, images, whiteboards, text etc.). For example, depending on the communication speeds available and the urgency of the communication, the interaction may consist of a completely asynchronous collaboration where two or more collaborators exchange diagnostic images via e-mail (or even by regular mail) and the diagnostic conversation can happen over days. The other extreme is where the collaboration is a real-time exchange of diagnostic images that is supported by video, audio and shared whiteboard and involves multiple collaborators around the world (such as can be supported by the free AccessGrid application) The existing communication infrastructure will ultimately dictate the level of interaction; however transmission speeds and coverage continue to increase on a regular basis. The wide availability of free e-mail services like Gmail and of open-source video conferencing environments further reduces the barriers for accessing these real time modes of internet enabled communication.

### An open and commoditized PACS

The development of the picture archiving and communication systems (PACS) needed to store this information was originally undertaken by large commercial concerns, such as GE Medical Systems. These were designed to store digital images of clinical scans and tests in a multi-user institutional PACS that could be linked to individual electronic medical records (EMR). The widespread use of PACS/EMR systems is creating a new media for collaboration within and between clinical departments as well as with researchers and patients that is rapidly being commoditized [21-23]. Technological barriers to linking different institutional PACS are rapidly being overcome. Furthermore, "open-source" systems are starting to appear and the concept of applying GRID computing (based on multiple networked computers) PACS solutions to the handling complex diagnostic challenges are being promoted [24,25]. In parallel, there is the potential to open up and commoditize the equipment specifications for institutional PACS solutions. Choong *et al *[26] examined how the cost of sharing x-ray images within a PACS in Malaysia could be reduced by using commodity solutions. They found that: 1) consumer digital cameras could digitize X-ray images to a standard that matched the expensive digitizers commonly specified for PACS solutions; 2) certain forms of "lossy" compression were well-tolerated enabling high data compression for more efficient data transfer; and 3) visualization of the images using an inexpensive consumer grade 15" LCD screen was just as effective as using an expensive high quality monitor in arriving at clinical decisions. This evidence implies that healthcare infrastructure is no different from any other form of measurement and communication infrastructure and can be subjected to commodity engineering. Experiences with virtual microscopy in the pathology sector indicate that the examination of virtual slides generally provide essentially the same diagnostic results as glass slides [6,13].

### Validation by example

**Figure 1:**
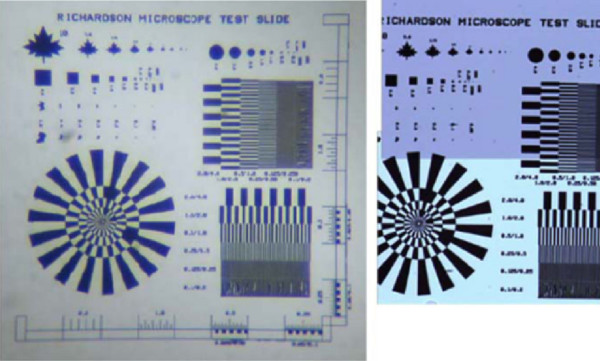
**Bright field images of Richardson Target Slide on two digital microscopy platforms**. The image of the Richardson test slide (US patent # 6381013) on the left was taken using a commodity system made up of an Intercase, Zeiss Primo Star microscope with a Canon A640 camera mounted on the trinocular port and a 100× 1.25 NA oil immersion objective. The image on the right was acquired with an Infinity 2 USB camera using proprietary Windows-based acquisition software. The camera was mounted on an Olympus BX41 clinical microscope which employed a 100× oil 1.4NA Plan Achromatic Objective. The Primo Star system and the Olympus system differed in price by 10 fold.

The feasibility of the informatics model presented above is validated by the example of designing, building and testing a complete LED based fluorescent/bright field telemicroscope workstation using commercially available components for under $2900 (plus access to a general purpose microscope; Table 1). Using this system, the results were as useful as those obtained from a much more expensive research grade photomicroscopy system. The ability to resolve details in a Richardson target slide with the two systems is comparable (Figure 1). Although contrast with the commodity system is poorer, no digital contrast enhancement methods where used with the commodity parts based system. The power of the computer used here allows the efficient utilization of a wide variety of digital algorithms for improving focus and contrast. This is an area for fruitful future research.

**Figure 2:**
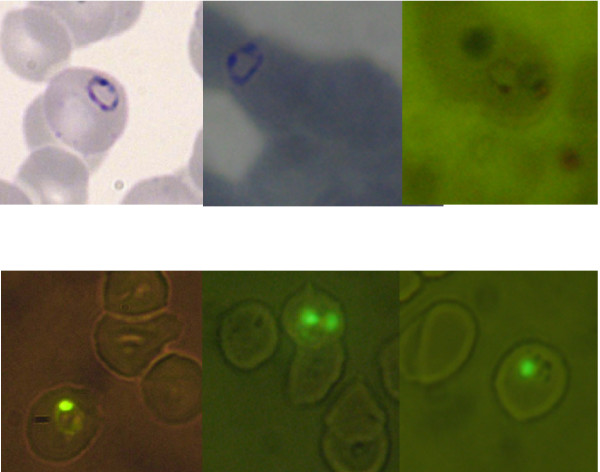
**Imaging of *Plasmodium falciparum *parasites using different microscopes**. The upper set of images represent typical bright field images obtained with: an Olympus BX41/Infinity 2 based digital microscopy system (left panel); a PrimoStar microscope using the 100× 1.0 NA oil immersion objective supplied with the microscope (middle panel); and the same PrimoStar fitted with a LW Scientific Lumin objective with 100× 1.25 NA oil immersion attachment (right panel). The specimen being imaged was a thin blood film prepared from a patient that had been diagnosed with *P. falciparum*. The lower set of images were obtained from methanol-fixed thin films which had been stained with SYBR Green I as described in reference 18. The image on the left was obtained using a Nikon Labophot Microscope equipped with a HB-10103AF epifluorescence attachment and the Infinity 2 camera system. The middle and right images were obtained using the PrimoStar fitted with the LW Scientific Lumin objective with a 100× 1.25 NA oil immersion attachment. Blue light (480 nm) was used to excite the SYBR Fluor and a low level of white light trans illumination was used to provide background detail.

Figure 2 illustrates the quality of imaging blood cells and the *P. falciparum *ring stage acquired using the commoditized system and a research digital microbiology microscope system sold by one of the big three microscope manufacturers. The top panel in 2 illustrates capacity to digitally record images of the ring stage in trans-illumination mode. The bottom panels illustrate the capacity to record images of parasites fluorescently stained with SYBR Green I and imaged both with epifluorescent blue light (480 nm) [17,18] from both a traditional and LED source. By using the gPhoto options to control point-&-shoot camera settings, reasonable contrast with the fluorescent image was obtained. Virtual slides for educational and quality assurance purposes can be assembled from such digital data [13].

The computational heart of the telemicrobiology workstation is a powerful, but "barebones", data processing and communication platform with associated peripheral devices and software. It is referred to here as an Intercase^©^, because it represents an engineered envelope encompassing both hardware and software; it is an integration of globally available commodity computer components and open-source software designed to link distributed diagnostic case events. Because the Intercase is implemented using commodity engineering principles it is supported by a globalized supply chain and a globalized network of software developers. The Intercase^© ^has sufficient computational power to meet the needs of most power users including advanced office productivity software as well teleconferencing applications and other forms of social networking media. When the Intercase^© ^is combined with simple microscopy hardware and a point-&-shoot camera, a local operator can view and capture microscopic images. These then can be annotated and shared with remote collaborators through various means including the Internet.

The authors have developed a new BioTIFF approach for storing large amounts of sequentially related diagnostic information. This format is an extended and indexable tagged image file format (TIFF) that accommodates additional metadata tags [27] so that all metadata needed to interpret the image based diagnostic data is in embedded in the image file itself. This approach greatly simplifies the task of creating registries and archives of the diagnostic events for both primary and secondary clinical and research uses. Furthermore, the data is easily stored and retrieved from data cloud services like the Amazon S3 storage cloud.

The Intercase^© ^used here runs a version of Linux that can support multiple languages (Ubuntu LTS 8.04 LTS) and is guaranteed to be a supported for several years through a large global developer community. The Intercase^© ^data processing platform is also designed to be almost completely solid state making it durable and energy efficient. The entire operating system resides on a password protected USB key. The Intercase is powered via a solid state DC/DC converter developed for the car-PC market by Powerstream Inc. (Orem, UT). This is driven by a commodity 12 V power transformer. Because electricity in many parts of the world is a very scarce resource, the Intercase^© ^is designed to be very energy efficient. Preliminary testing indicates an Intercase^©^, a LCD monitor, a microscope (including operating LED light source for trans- and epifluorescence illumination) all together use approximately 60 watts. Because it is relatively power efficient, a rechargeable battery-based UPS can be used to protect against irregular electricity supplies and provide a receptor for diverse power generating strategies.

Any container can be used as a computer case; in the example presented here a simple plastic food storage container is used. A tested components list for the TeleMicrobiology Intercase is provided in Table 2. It is anticipated that many other substantially equivalent alternative component lists are possible and in many cases will be more appropriate for specific locales.

**Table 2:** TeleMicrobiology Intercase Parts Lists ($2,900)

*1-Sample Illuminator/Interrogator *($1,700)	*4-Data processing Software *(Free)
• 100×, 1.0NA LW Lumin Objective	• Operating System (BioTIFF Ubuntu 8.04 LTS)
• LW Lumin LED Illuminator	• Data acquisition, (gPhoto)
*2-Digital sensor *($600)	• Collaboration software (BioTIFF-Access Grid)
• Canon A640 digital point-&-shoot camera^1^	• Annotation software (BioTIFF 1.0)
• Canon LA-DC58F, 58 mm Conversion Lens	• Intercase Network Interpretation Services
• Zeiss Digital Camera Adapter P95 M37/52 × 0.75	• Reporting software (Open Office, gMail, Firefox)
*3-Data processing Hardware *($300)	*5-Data/Image Display ($200)*
• commodity motherboard/CPU combo	• 14", 1024 × 728, 13 Watt (MegaVision MV142)^1^
(MX1333 motherboard/Pentium 4 E-2160 CPU)^1^	*6-Power/cooling/enclosure components *($100)
• RAM (Kingston 2 GB)	• Power Kit (Pico PSU 90 Power Kit)
• Live Operating System Key (8 GB Cruzer)	• CPU Fan
• USB Hub, keyboard, mouse speakers, microphone	• Intercase Box

^1^Part no longer available but can be source used or replaced by equivalent next generation part.

## Discussion

In this methodology paper, an informatic model was developed for sharing microbiological information using *ad-hoc *assemblies of commodity components and open-source software and validated the feasibility of implementing this model using the challenge of routine microscopy based-diagnosis of malaria as an example. This paper demonstrates that there are no technical barriers limiting the introduction of this approach into clinical microbiology laboratories in either low- or high-income countries. Widespread use of technologies to transmit images and other data in combination with increasing familiarity with web-based social networking sites may lead to tolerance of greater equipment diversity while achieving better quality diagnostics, particularly in resource-poor environments. Significant issues surrounding the adoption of collaborative diagnostic technologies exist, including regulatory issues and potential resistance of laboratory workers and managers to change their practices and to tolerate performance criticisms from afar. However, the uncertainty of change may be balanced by the potential for greater individual recognition and demonstrable capacity for autonomy as well as the opportunity to participate in grassroots efforts to address global health challenges, thereby creating greater job satisfaction and attracting new recruits to a winnable struggle.

The generic and open telemicroscopy technology described here can address several widely recognized clinical microbiology infrastructure deficits in an economically sound and sustainable manner (see Table 1). It can boost capacity to deal with comprehensive measurement of disease and care outcomes in individuals and groups in a reciprocal and collaborative fashion. The commodity collaborative workstation model enables local control over the creation and use of laboratory medicine diagnostic infrastructure, while enabling remote collaborative support for diagnostic interpretation. It can also enable global pooling of malaria disease information and the development of open, participatory, and adaptable laboratory medicine practices. Generic diagnostic platforms built of commodity elements are by definition transparent and open, and therefore "regulation friendly". The networked functionality allows for distributed oversight over performance since data generated can be tracked, transmitted, and analysed at a central location. This model facilitates external quality assessment (EQA) programmes essential for ensuring that diagnostic system work as specified after being established [1,13]. In addition, participation in a networked diagnostic system allows access to other non-local individuals who can be called upon to provide mentorship and interpretive oversight. Conversely, home-grown expertise and innovation can be more easily recognized and shared. Finally, infrastructure developed to serve a global health need to track one disease like malaria can also be used to support other routine microscope-based laboratory medicine applications like haematology, microbiology, and parasitology services.

Collaboratively designed open systems, particularly those based on commodity components, will make visible not only how the diagnostic test is performed, but also the components needed to implement a diagnostic process. This transparency enables the performance of a particular diagnostic test in a particular setting to be evaluated and validated, while still allowing for adaptation to local conditions or changes in the local technology ecology as well as natural selection of useful innovations. This in turn creates the potential for optimization of diagnostic processes by the end-users themselves since they can examine the underlying design and code and improve upon them. Proprietary systems frequently lack transparency since "black boxes" which take input(s) and provide output(s) in ways guarded by licenses, patents, and trade secrets are usually dependent on tightly managed service channels and are often not accessible in low-income countries, let alone encouraging of accommodating local needs.

Networking has potential benefits that extend beyond individuals' professional development and local performance quality improvement, as has been demonstrated by the GeoSentinel Network. The purpose of the GeoSentinel Network is to spot disease trends and emerging infections by tracking case reports [28]. Direct ongoing analysis of local drug response and resistance patterns could support surveillance of the emergence of drug resistant malaria strains [29] which can have dramatic consequences for health care delivery and expenditures [30,31].

In 2003, a Grand Challenges in Global Health (GCGH) investment fund was set up by the Bill and Melinda Gates Foundation. This fund commissioned an international committee of experts to identify a limited set of seven goals likely to provide considerable returns of investment in technology development and to identify fourteen Global Health Grand Challenges (GHGCs), defined as problems where significant investment and development efforts aimed at creating solutions would have the most promise of having a major impact on global health within a few years [32]. A major goal they identified was "to measure disease and health status accurately and economically in poor countries" and this was further linked to two major Grand Challenges: "GHGC 13 – develop technologies that permit quantitative assessment of population health status; and GHGC 14-develop technologies that allow assessment of individuals for multiple conditions or pathogens at point of care". The preceding discussion points to ways in which these two GHGCs can be addressed. However, in contrast to the "best-of-the-best" new product and "overwhelming force" attitudes implicit in the GCGH approach, the model presented here illustrates how the needed technologies already exist in the consumer electronic markets of the world and in the nimble communication networks that are providing services to the bottom of world's economic pyramid [33]. Rather than focusing on emulating the regime of highly engineered, highly regulated (and often 'sub-prime') single purpose health technologies characteristic of health care technology markets in high-income countries, the framework suggested here could allow diagnostic devices to be economically assembled and operated locally using local expertise. This approach will be more easily adapted to local needs and will encourage multi-vendor competition.

The laboratory medicine deficits hindering disease control and surveillance efforts in low income countries have direct correlates with deficits evident in high income countries such as Canada [34]. Thus, innovation initiated by low-income countries to overcome infrastructure and human resource limitations have the potential of helping high-income countries escape from increasingly unsustainable approaches to health care delivery. It can be estimated that currently more than 10% (or >$7 trillion) of the global GDP is consumed in the healthcare sector with far from satisfactory results [35,36]. Most of this global expenditure is directed at dealing with health care concerns of the richest 10% of the global population.

Much of the failure to deal with laboratory infrastructure deficits in both low- and high-income countries is a social-technical issue that cannot be overcome simply though technological or fiscal innovations [37]. More than half the world's population is living on less than $2.50 day and 25% of the world's population make between $1.25 and $2.50 [38]. Thus, although the latter groups spends a significant proportion of their earning on dealing with chronic diseases their capacity to spare hard earned capital to purchase North American style clinical services at North American prices is extremely limited. Simplistic market economy solutions will not directly deal with their needs [38]. Nevertheless, the popularity, wide dissemination, and ease of use and reuse/repurposing of commodity consumer electronic, communication, and computation technology, that is accessible even in low-income countries [33], provides the means to build and maintain adaptable and scalable health informatics systems. The missing ingredient for enabling such a transformation is a framework for guiding establishment of substantially equivalent alternative systems that can be built up in a sustainable manner. This paper outlines one such framework.

## Conclusion

With barriers to accessing the Internet falling around the world, issues of local standardization, calibration, and quality assessment that are at the core of delivering reliable diagnostics [3,4] may now be more easily addressed globally. At the core of any diagnostics system is an informatics model that guides the specification of equipment required to mediate desired flow of information. Health systems in general, whether local, national, or international can be considered as primarily a means for organizing access to trusted and reliable expert knowledge [39]. This paper has presented a method for establishing a telemicroscopy system for malaria surveillance that can not only help in the global quest to "Roll Back Malaria" but also provide a means for delivering other forms of microscopy-based clinical laboratory procedures locally. An ability to generate and share high definition virtual slides of interesting or problematic samples can allow for the development of distributed communities of practice that can raise global health capacity globally. Distributed digital image-based telemicrobiology networks deployed for dealing with malaria can serve as templates and levers for assuring access to trusted and reliable diagnostic information anywhere.

## Competing interests

The authors are also principles in a R&D corporation called gDial Inc. that is developing a commercial implementation of the ideas described in this paper.

## Authors' contributions

WS conceived of the technology framework, and developed the software and hardware Intercase platform. IC provided samples and microbiological insights into the challenge. He carried out all of the preparation of microbiology samples. PP wrote the bulk of the manuscript with major input from the other two authors. All authors have read and approved the final manuscript.
